# Impact of legal status regularization on undocumented migrants’ self-reported and mental health in Switzerland

**DOI:** 10.1016/j.ssmph.2023.101398

**Published:** 2023-04-06

**Authors:** Jan-Erik Refle, Julien Fakhoury, Claudine Burton-Jeangros, Liala Consoli, Yves Jackson

**Affiliations:** a LIVES, Swiss Centre of Expertise in Life Course Research, University of Geneva, Geneva, Switzerland; b Center for the Interdisciplinary Study of Gerontology and Vulnerability, University of Geneva, Geneva, Switzerland; c Institute of Sociological Research, University of Geneva, Geneva, Switzerland; d Division of Primary Care Medicine, Geneva University Hospital and University of Geneva, Geneva, Switzerland

**Keywords:** Health, Mental health, Depression, Migrants, Undocumented, Regularization, Vulnerability, Longitudinal

## Abstract

Undocumented migrants face cumulative difficulties like precarious living and working conditions or exclusion from health services that might negatively influence their health. Little is known about the evolution of undocumented migrants’ self-reported health (SRH) and mental health after they get documented. This study aims to observe the effect of legal status regularization on SRH and mental health in a cohort of migrants undergoing regularization in Geneva, Switzerland. We evaluate SRH with the first item of the Short Form Survey (SF12) and depression as a proxy of mental health with the PHQ-9 questionnaire over four years among 387 undocumented and newly documented migrants. Using hybrid linear models, our data show that regularization has no direct effect on SRH, but has direct positive effects on mental health in a longitudinal perspective, even when controlling for competing factors. The arrival of the pandemic did not alter these effects. Migrants tend to evaluate their subjective health status more positively than the prevalence of screened depression shows. Those findings point towards better targeted policies that could reduce the burden of depression among undocumented migrants.

## Highlights

• Regularization positively influences mental health.
• The process of regularization does not directly alter self-reported health but regularized migrants report better health.
• We observe a discrepancy between self-reported health and depression among undocumented migrants.

## 1 Introduction

Undocumented migrants account for between estimated 2 and 4 million people in Europe and 76′000 in Switzerland with exact numbers being unclear due to different legal status definitions (Vogel et al., 2011; Morlok et al., 2016). The living conditions of undocumented migrants are precarious due to the multiplicity of hardships encountered simultaneously in the absence of basic securities. The lack of residence permit notably exposes to abusive working conditions in the informal labor market, to barriers in the access to healthcare and accommodation, to restricted mobility as well as to the constant fear of being deported (Benach et al., 2011; Consoli et al., 2022). Considering these factors, the self-reported health status (SRH) of undocumented migrants is generally poorer compared to the general population (Bombak & Bruce, 2012). In addition to self-reported health, previous studies observed that undocumented workers are struggling more with mental health issues, especially in comparison to documented workers, but also in comparison to the general population (Fakhoury et al., 2021; Teunissen et al., 2014; Heeren et al., 2014). However, most studies rely on qualitative or cross-sectional quantitative data due to problems in engaging with and following across time this hard-to-reach population. Due to the scarcity of data on undocumented migrant populations, we know relatively little about what happens to self-reported as well as mental health during a process of residence status regularization. Up to our knowledge, there is no study that showed the direct effects of regularization on health over a several-years’ timeframe that includes the moment of regularization in a European context. We thereby formulate the following research question: What are the effects of regularization on migrants' self-rated health and mental health in a longitudinal perspective?

In order to address this question, we used unique panel data that followed migrants undergoing regularization through an exceptional program – the “Papyrus Operation”- implemented in the Swiss Canton of Geneva. Between 2017 and 2021, migrants were surveyed over four waves before, throughout and after regularization and their self-reported health and mental health were assessed at each time point. Most of the studies on undocumented and newly regularized migrants are cross-sectional or do not follow the same persons over time. As recent literature reviews found (Winters et al., 2018), panel studies on undocumented and newly regularized migrants are nearly non-existent due to the above-mentioned reasons. One of the rare examples is the Mexican Migration Project (MMP; Thom, 2010) and other studies on labor market data from the U.S. (Bratsberg et al., 2002). Up to our knowledge, there is no other study using panel data in a European country on newly regularized and undocumented migrants.

The period of study included the emergence of the COVID-19 pandemic in 2020, giving us the opportunity to assess how such a major event could potentially influence their health. In our study, we used the self-reported health indicator (Wurm & Benyamini, 2014) as a subjective evaluation of migrants’ own health. Complementarily, we used the Patient Health Questionnaire-9 (PHQ-9) score that screens for depression severity (Kroenke et al., 2001; Kroenke & Spitzer, 2002). We estimated hybrid models with SRH and PHQ-9 as dependent variables.

Our contribution includes four elements: first we assess the effects of regularization on self-reported health and mental health. Second, we not only analyze the effect of regularization, but we also control for other effects that derive from regularization, like changes in the housing situation, thereby controlling whether regularization effects are robust once controlling for its indirect effects. Third, we bring in knowledge on the effects of regularization in a European and more concretely Swiss context. As most studies currently concern the U.S., our contribution is important for advancing research and better understanding the living conditions of undocumented and newly regularized migrants in Europe. Fourth, we address the relationship between regularization and health in an exceptional period, by specifically testing whether the Covid-19 pandemic had a negative effect on undocumented migrants' SRH and mental health. In the following we first review literature on migrants’ SRH and mental health as well as the Covid-19 related influences before deriving hypotheses. In the onset, the background of the study and methodological considerations are discussed before we expose our results.

### 1.1 Migrants' self-reported health

We use the subjective self-evaluation of one's health as the first dependent variable likely to be influenced by residence status. Many studies on migrants and self-reported health concern access to healthcare and how this access influences evaluations (Dorn et al., 2011; Magalhaes et al., 2010; Schoevers et al., 2009; Sebo et al., 2011; Teunissen et al., 2014; Winters et al., 2018). For instance, some studies underscore that different government policies strongly influence migrant health with restrictive policies negatively influencing their healthcare opportunities (Juárez et al., 2019; Lamkaddem et al., 2015). As undocumented migrants face exclusion from a number of healthcare services, it is argued that they cannot afford necessary medical care, avoid care due to the risk of denunciation or are simply not aware of care possibilities (Malhahaes et al., 2010; Woodward et al., 2014). Poorer self-reported health among undocumented migrants is associated with hardworking conditions, limited social interaction as well as precarious living conditions (Ahonen et al., 2009; Fakhoury et al., 2021; Jolivet et al., 2012; Kumparatana et al., 2017; Pikhart et al., 2010; Sousa et al., 2010; Woodward et al., 2014). This could explain the higher mortality rates among undocumented migrants recently reported in Switzerland (Piccoli & Wanner, 2022). However, scholars showed that the difference in health evaluations is not primarily due to the acquisition of a residence status, but results from better healthcare and socioeconomic opportunities once documented (Cloos et al., 2020). Socioeconomic characteristics such as income levels or work situation are important determinants that are found to explain SRH among the general population (Castañeda et al., 2015; Cullati et al., 2014). Nevertheless, studies assessing differences between undocumented migrants and newly regularized ones are scarce. As a consequence, we know relatively little about when changes in health should be expected, i.e., whether changes in SRH happen directly after regularization – for example due to immediate access to healthcare – or later on – for example as a result of improved living conditions over a longer time.

### 1.2 Mental health of undocumented migrants

Depression is identified as one of the main health problems of undocumented migrants (Winters et al., 2018; Woodward et al., 2014). However, it remains unclear whether migrants’ mental health mainly deteriorates after settling in the destination country or is mostly attributable to exposures to health risks before or along the migration journey, as seen in asylum seekers (Chen et al., 2017; Wu et al., 2021). For instance, some findings indicate that directly after migration, migrants might display good mental health due to initial hopes to realize aspirations (Bhugra et al., 2011). In addition, they often perceive their status as undocumented as something temporary that is unlikely to persist (Consoli et al., 2022). Still other researchers underscore that arriving migrants may have experienced traumas in the country of origin, providing an additional motivation for migration (Torres et al., 2018), leading to an overall divided picture.

Existing studies show a higher burden of depression, more stressors and less health service consumption among undocumented migrants (Garcini et al., 2017; Levitt et al., 2019; Dillon et al., 2018; Sudhinaraset et al., 2022). In general, most studies apply to the U.S. context due to better data availability. Although undocumented migrants are a very heterogeneous group whose socio-demographic characteristics variate from one context to another, studies led in Europe point to high prevalence of mental disorders (Andersson et al., 2018). For instance, studies in Switzerland estimated the prevalence of symptoms of anxiety and depression among undocumented migrants at around 45% and 50% respectively, proportions that are much higher than those observed in the general population (Heeren et al., 2014; Fakhoury et al., 2021). In their systematic reviews, Garcini et al. (2021) as well as Vernice et al. (2020) show that undocumented migrants face exclusion from health services while experiencing high levels of fear and anxiety at the same time. Similar to SRH, literature also confirms the link between stressors that influence migrants’ mental health status and the level of restrictive policies in a country or area (Torres et al., 2018).

Additional literature on migrants in general showed similar tendencies and nuanced variations across sub-populations. Indeed, studies concluded that mental health scores differ not significantly among ethnic groups, but across generations of migrants in a country of destination (Tibubos et al., 2018). Literature on migrants and health also shows, in the U.S. as well as in European contexts, that mental health services utilization differs between ethnic groups of migrants (Lindert et al., 2008; Vega et al., 2001).

Cross-sectional data suggested positive effects of regularization on mental health. More specifically, in the context of the Deferred Action for Childhood Arrivals (DACA) implemented in 2012 in the U.S. and targeting young undocumented migrants eligible for regularization, studies found associations between legal status regularization and reduced mental distress (Venkataramani et al., 2017; Patler & Pirtle, 2018). Similar findings apply to Canada (Magalhaes et al., 2010). In Switzerland, newly regularized migrants also displayed a lower prevalence of mental health conditions than undocumented ones (Fakhoury et al., 2021). However, the direct association between legal status regularization and better mental health revealed insignificant after adjustment for various measures of social support, integration and economic resources. To our knowledge, no panel study has aimed at evaluating the impact of regularization on mental health in a longitudinal perspective. While we know that mental health of documented migrants is better compared to undocumented migrants, we do not know whether this difference is resulting from regularization, nor when this change happens.

### 1.3 COVID-19 and its intervening effect during the study

Migrants, especially those undocumented, were particularly impacted by the pandemic, as it worsened already existing economic and social pressures (Burton-Jeangros et al., 2020; Mengesha et al., 2022; Van denMuijsenbergh, Torensma, Skowronek, de Lange, & Stronks, 2022; Kumar et al., 2021; Greenaway et al., 2020). Qualitative research shows that undocumented migrants faced insecurity in numerous life domains that were more pronounced due to their already vulnerable position (Van denMuijsenbergh et al., 2022). Among others, higher infection rates, due to higher housing density, as well as higher mortality and more negative economic impact are some of the consequences for migrant populations (Greenaway et al., 2020; Kumar et al., 2021). Furthermore, research has shown that migrants’ mental health was negatively impacted by the consequences of the pandemic (Marchi et al., 2022). The notion of syndemic has been associated to the COVID-19 pandemic, to account for these interactions between the virus and pre-existing social and health inequalities (Bambra et al., 2020). Based on this literature we expect that the pandemic, concomitant to our data collection from wave 3 on, could have had the same negative impact on self-reported health and mental health of undocumented as well as regularized migrants. As the pandemic broke out just after the third wave data collection and extended until the end of the fourth wave, we are interested in assessing whether the effects of regularization are robust even in case of an unforeseeable external event.

### 1.4 Hypothesis

From the literature, we expect to observe better SRH among regularized compared to undocumented migrants and we equally expect a positive development over time once regularized. A positive development is expected to result from increased access to healthcare services and improvements in socioeconomic conditions. Concerning depression (measured through PHQ-9), we expect lower levels among regularized compared to undocumented migrants as well as a positive effect of regularization towards lower depression severity over time due to stressors getting reduced as access to state services increases and work and social situation stabilize. In line with the evoked literature, we expect that due to a generally better life situation, depression and the feeling of being blocked diminish after regularization.

Regarding the effect of the pandemic, we expect independent effects of the pandemic on both SRH (lowering SRH) and depression as the pandemic increased many existing problems among migrants. As a result, we formulated the hypothesis *that becoming regularized was associated with better self-rated health and mental health, notwithstanding the effects of the pandemic*, which is expected to negatively affect self-rated health and mental health.

## 2 Methodology

### 2.1 Setting

This paper is set in the context of a unique regularization initiative – the “Papyrus Operation” - implemented in the Swiss Canton of Geneva between 2017 and 2018. This initiative foresaw that migrants that came neither from European Free Trade Area (EFTA) nor 10.13039/501100000780European Union (EU) countries and who could prove a certain income and having lived in Geneva for at least 10 years (5 years if having children at school) together with other requirements such as basic language skills, could demand their regularization (rejected asylum seeker had no chance to get granted regularization). The program led to the regularization of about 2390 formerly undocumented migrants. Concomitantly, the Parchemins Study accompanied a share of those migrants as well as undocumented migrants who could not apply for regularization, as part of a mixed method design with qualitative and quantitative panel data collected over four waves between 2017 and 2021 (Jackson et al., 2019). Our paper uses the data generated through this study. Besides not being a national of a country member of the EFTA or the EU, the Parchemins study sat three additional participation criteria: being aged 18 or more, having never sought asylum in Switzerland and having lived without discontinuation for at least 3 years in Geneva. These criteria aimed at selecting a control group with similar background compared to those that got regularized.

Participants were recruited via associations active on migrant rights (about 75% of the sample) and at a dedicated medical center where uninsured migrants can consult free of charge (for a detailed overview on recruitment, we refer to Jackson et al., 2019). Both recruitment channels ensured a trusted relationship from participants who gave informed written consent to their participation. Collected data include a life calendar until migration, a survey questionnaire and - for some individuals - qualitative in-depth interviews. The questionnaire was administered face-to-face in French, Spanish, Portuguese, or English; during the COVID-19 pandemic data was collected digitally. The study protocol was approved by the Ethics Committee of the Canton of Geneva, Switzerland (authorization 2017–00897).

### 2.2 Participants

**Fig. 1:**
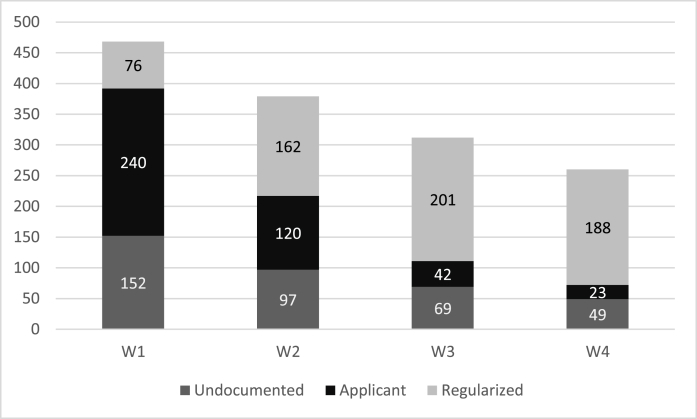
Number of participants by wave and residence permit status Note = ‘regularized’ includes those who already got a permit, ‘applicant’ those who started the application procedure, ‘undocumented’ those that did not apply. Data source: Parchemins study.

In this article, we focus on the quantitative part of the Parchemins-study that surveyed 468 migrants and assessed their general and mental health status over four waves. The number of participants and their status by waves is found in Fig. 1 (for a full panel description: Jackson et al., 2022). Similar to other panel studies and as result of respondents being part of a hard-to-reach population, attrition was relatively high across the four waves. Indeed, undocumented migrants are hard to recruit because of their unstable status and migrants have a higher drop-out rate to panels in general (Rothenbühler & Voorpostel, 2016; Lipps, 2007). However, the study deployed an intense effort in terms of time and resources to reach the respondents over time. This means, that participants have been (re-) contacted at different hours and days, via different contact ways (e.g. calls, supplemented by mails and WhatsApp), and with a high number of contact trials. Without the additional efforts deployed, participation rates in computer-assisted interviews would have much lower.

### 2.3 Variables

Residence status regularization is our main exposure of interest and was measured repeatedly in every wave. It is a nominal variable with three categories. First, we distinguish between those who applied for regularization but were waiting for a legal decision and those that did not engage in the regularization process. This distinction reflects the possibility already offered to regularization applicants to access to some public services while waiting for the legal decision, although most benefits depend on being officially regularized. Thus, once participants filled an application, they were included in the category “applicant”. Second, if their application was approved, they were moved to the category “regularized”. For instance, a participant could change from “undocumented” to “applicant” and then to “regularized” across the four waves. Given the specific configuration of the regularization program, with community associations serving as gatekeepers that helped fill the applications, the number of rejections was very limited among those that applied. As a result, changes from “applicant” to “undocumented” among participants were scarce. On the other hand, due to administrative delays, the regularization procedure could take up to 18 months, leading to participants remaining “applicant” for two or more waves. Detailed descriptions on the panel and its characteristics are found in Jackson et al. (2022).

We test two dependent variables: self-reported health and depression. SRH is used to assess the subjective evaluation of one's own health. Respondents were asked to evaluate their health using a 5-point-scale, with 1 equaling poor health and 5 equaling excellent health (item1 of Short Form Survey (SF-12); Hays et al., 1998). The item is an established measure for the auto-evaluation of health in international surveys due notably to its consistent association with mortality (Wurm & Benyamini, 2014; Barofsky et al., 2004; Cullati et al., 2020; Idler & Benyamini, 1997; Sousa et al., 2010). Still, research equally showed that SRH and objective measures of health do not always coincide and that minority groups report generally lower levels of SRH (Bombak & Bruce, 2012). Similarly, SRH and depressive symptoms are correlated, but the causal direction as well as whether for example age plays a role are disputed (Peleg & Nudelman, 2021).

Mental health was assessed using depression as a proxy since preliminary results of the Parchemins study showed high prevalence of depression among undocumented and regularized migrants in a cross-sectional perspective (Fakhoury et al., 2021). Depression was measured using the patient health questionnaire (PHQ-9) score. It is based on an additive scale derived from nine items (Kroenke et al. 2001, 2009; Kroenke & Spitzer, 2002) and the total score ranges from 0 to 27 points. Scores equal or above 5 indicate the presence of depressive symptoms and the higher the score, the more severe the symptoms of depression. The list of the items is found in the annex.

### 2.4 Confounding and control variables

In line with literature on health status, we check whether regularization over time has an effect, regardless of whether documented migrants report overall higher SRH and expose better mental health levels than undocumented ones. Nonetheless, residence status regularization in the framework of the Papyrus Operation was selective and based on several criteria that could theoretically influence health. We thus include the following confounders: the length of stay in Geneva at baseline, the ability to pay a CHF 1500.- (€1500.-; $1570.-) unexpected bill (No vs. Yes) as a marker of economic precariousness, the number of working hours per week and the self-assessed oral proficiency in French (Good to excellent vs. Very bad to sufficient). These potential confounders, except the length of stay in Geneva at baseline, could variate at the individual level from one wave to the next. As the oral proficiency in French was not measured in the second wave, we imputed the first wave responses. Regarding the ability to pay an unexpected bill, CHF 1500.- is a large amount in many countries, but it represents less than one fourth of the average monthly salary in Geneva. For comparison, the average monthly income in wave 1 was at roughly 2500 CHF among our sample, wherefrom 1500 CHF equal thereby 60% of the income.

Age, sex, origin (Latin America, Africa, East Asia, Eastern Europe), level of education (primary, secondary, tertiary), civil partnership status (single, unmarried couple, couple), the number of children and the satisfaction with the housing conditions (scale from 0 to 10) are added as control variables. Similar to some of the confounders, participants’ partnership status, number of children and satisfaction with the housing conditions could change over time.

Our data collection fell partially in the time of the COVID-19 pandemic. Thus, in order to verify a possible period effect, we test in our models whether there are significant differences between wave 3, collected during the first stages of the COVID-19 pandemic, and the other data collection waves.

### 2.5 Data and measurement

Categorical data are described as absolute numbers and relative percentages (%), continuous ones as means, standard deviations (SD), minimum value (min.) and maximum value (max.). To assess whether regularization led to a change in SRH and PHQ-9 scores, hybrid linear models are used. Hybrid models are particularly useful, as they allow to disentangle the within-individual from the between-individual (i.e., group-level) effects of the time-varying variables. Hybrid models also benefit from the advantages of fixed effect models, as they consistently estimate the within-individual variance over time, while similarly allowing taking into account time-invariant characteristics. With time-invariant characteristics, we describe all those individual characteristics that do not or only rarely change over time, like the origin of respondents for example.

This study aims to distinguish the within-individual effect from regularization – that is, the changes in SRH and PHQ-9 associated with regularization at the intra-individual level – from the between-individual effect of regularization. This distinction allows isolating the direct effect of regularization on individuals while controlling for other variables that may differ when comparing undocumented and regularized migrants. To put it differently, differences in health could be partly explained by group-level differences, that is by differences between undocumented and regularized migrants, while within-subjects effects would reflect the variance in individuals’ health that is due to their regularization.

We present two models for each outcome (SRH and PHQ-9 score). The first model includes the within-individual effect of regularization, the between-individual effect of having a residence status and the data collection waves as proxies for period effects. The second model is adjusted for all confounders and control variables presented in the above section. While the first one shows how much variance is explained by the variables of interest only, the second model controls for confounders and whether increased health levels and altered mental health are influenced by other socio-economic factors.

The sample included individuals (level-2 units) with at least two observations (level-1 units). All models were estimated in R (version 4.0.3) using lme4, lmerTest and panelr packages.

## 3 Results

### 3.1 Participants sociodemographic characteristics

The final sample consisted of 1304 observations clustered within 387 participants. Participants were predominantly women (74%) and the majority had pursued secondary education at least (91.2%) (Table 1). On average, participants had been living in Geneva for 12 years when the Operation Papyrus was implemented. Latin America (66.4%) and East Asia (21.1%) represented the main regions of origin.

**Table 1:** Participants characteristics in wave 1.

Variables	Total (n = 387)
	Mean ± SD (Min-Max) or Number (% total, *% undocumented/%applicant/% regularized*)
Sex	
*Female*	286 (73.9%, *76%/71%/79*%)
*Male*	101 (26.1%,*24%/29%/21%)*
Origin	
*Latin America*	257 (66.4%/*66%/62/81%)*
*Africa*	22 (5.7%, *7%/5%/5%)*
*East Asia*	82 (21.1%, *24%/24%/11%)*
*Eastern Europe*	26 (6.7%, *4%/10%/3%)*
Age	43.9 (*41/45/45*) ± 11.0 (18–73)
Level of education	
*Primary*	34 (8.8%, *8%/8%/11%)*
*Secondary*	264 (68.2%, *65%/70%/68%)*
*Tertiary*	89 (22.9%, *26%/22%/21%)*
Length of stay in Geneva at baseline	12 (*8/13/14*) ± 5.2 (3–37)

### 3.2 SRH and mental health

Measures of SRH and depression (Table 2, Table 3) show different developments over time. Levels of SRH fluctuated slightly around an average value of 3.3 across all waves, with no perceptible impact of the COVID-19 pandemic in Wave 3. PHQ-9 score augmented during the pandemic (from wave 3 onwards). The in-average one point increase is also found in other studies examining the effect of the pandemic on the general population's mental health (Peters et al., 2020).

**Table 2:** Descriptive statistics for the dependent variable SRH a

	N	Mean	SD
Self-Rated Health (Wave 1)	378	3.25	.95
Self-Rated Health (Wave 2)	365	3.34	.91
Self-Rated Health (Wave 3)	304	3.30	.93
Self-Rated Health (Wave 4)	257	3.30	.86

Note: Scale from 1 (poor) to 5 (excellent) *Data source: Parchemins study*.

a SRH at baseline is at 2.9 (SD: 1.0) for undocumented, 3.4 (0.9) for applicants and 3.5 (0.8) for regularized.

**Table 3:** Descriptive statistics for the dependent variable PHQ-scale, severity of depressive disorder - score.^a^.

	n	Mean	SD
PHQ-9 (Wave 1)	378	5.43	4.77
PHQ-9 (Wave 2)	365	5.52	5.20
PHQ-9 (Wave 3)	304	6.20	5.51
PHQ-9 (Wave 4)	257	6.29	5.21

Note: scale from 0 (no depressive symptoms) to 27 (severe symptoms) *Data source: Parchemins study*.

a PHQ at baseline is at 7.2 (SD:5.5) for undocumented, 4.9 (4.5) for applicants and 4.4 (3.7) for regularized.

### 3.3 Factors associated with SRH and mental health

In the following, we turn to multivariate hybrid models in order to explain changes in self-rated health and mental health over time. We first present findings for SRH, comparing the general quality of the models and then commenting on the relevant hypotheses.

When resuming findings from Table 4, we first observe the differences in inter and intra-individual variance that is explained by both models. The quality of model 1 including only regularization and wave's influence on SRH is limited with 6% of the interindividual variance explained. Explained residual variance accounts towards zero when using SRH as dependent variable, meaning that there are quasi no changes in SRH over time related to the variables included in the model. In practical terms that means that becoming regularized as an individual does not alter SRH levels afterwards. Model 2 adds much more interindividual variance, reaching 20%, but residual variance over time stays quasi-unchanged.

**Table 4:** Effect of regularization on SRH using hybrid models.

	SRH	
	Model 1	Model 2
	B coeff (95% CI)	B coeff (95% CI)
Applicant (ref. undocumented) (within-individual effect)	0.19 (−0.00, 0.38)	0.16 (−0.04, 0.35)
Regularized (ref. undocumented) (within-individual effect)	0.06 (−0.16, 0.28)	0.02 (−0.21, 0.24)
Applicant (ref. undocumented) (between-individual effect)	0.32 (0.07, 0.57)*	0.21 (−0.04, 0.46)
Regularized (ref. undocumented) (between-individual effect)	0.46 (0.26, 0.67)***	0.34 (0.13, 0.55)**
Wave 1 (ref. Wave 3)	−0.06 (−0.19, 0.01)	−0.08 (−0.20, 0.04)
Wave 2 (ref. Wave 3)	0.04 (−0.07, 0.15)	0.02 (−0.09, 0.12)
Wave 4 (ref. Wave 3)	0.01 (−0.11, 0.12)	−0.00 (−0.12, 0.11)
Women (ref. Men)		0.11 (−0.06, 0.28)
Age at baseline		0.00 (−0.01, 0.01)
African (ref. Latino American)		−0.24 (−0.54, 0.07)
East Asian (ref. Latino American)		0.08 (−0.09, 0.25)
Eastern European (ref. Latino American)		0.22 (−0.09, 0.53)
Secondary education (ref. Primary education)		0.32 (0.08, 0.57)*
Tertiary education (ref. Primary education)		0.22 (−0.06, 0.49)
Length of stay at baseline		−0.00 (−0.02, 0.02)
Ability to face an unexpected bill (ref. No)		0.2 (0.10, 0.31)***
Number of working hours		0.003 (0.000, 0.01)*
Satisfaction with housing conditions (scale from 0 to 10)		0.03 (0.01, 0.05)**
Poor (very bad to sufficient) proficiency in French (ref. Good to Excellent)		−0.03 (−0.28, 0.02)
Partnership status (single) (ref. Married)		−0.13 (−0.28, 0.20)
Partnership status (unmarried couple) (ref. Married)		−0.07 (−0.22, 0.09)
Nb. Children		−0.03 (−0.08, 0.02)
Observations (n, N)	387, 1304	387, 1304
R2 interindividual	0.06	0.2
R2 residual	0.00	0.00

***p < 0.001; **p < 0.01; *p < 0.05, Data source: Parchemins study.

In Model 1, regularization is significantly associated with SRH at the between-individual level: overall, regularized migrants (B = 0.46; 95% CI: 0.26, 0.67) and migrants who applied for regularization (B = 0.32; 95% CI: 0.07, 0.57) report higher levels of SRH compared to undocumented migrants. On the other hand, no direct within-individual effect of regularization is observed, meaning that at the individual level, being granted regularization has no direct impact on SRH (B = 0.06; 95% CI: 0.16, 0.28). The onset of the pandemic on Wave 3 is not associated with any effect on SRH.

After adjustment for socio-economic and socio-demographic factors in Model 2, regularized migrants still display better SRH compared to undocumented ones (B = 0.34; 95% CI: 0.13, 0.55). In addition, education, the ability to face an unexpected bill (a proxy for a precarious income situation), working hours and satisfaction with housing conditions have significant impact on SRH. This finding underscores the importance of socio-economic variables for explaining levels of SRH. However, at the within-individual level, the association between SRH and regularization remained insignificant. The general finding that documented migrants report better SRH might be linked to the selection process caused by the requirements of the regularization program (Fakhoury et al., 2021). As pointed out earlier, the regularization procedure was bound to specific requirements such as having a certain income. As outlined under 2.1, this can have influenced the levels of SRH among our population, as the group of participants that become regularized faced already higher work stability beforehand. This higher work stability includes a generally better situation in terms of SES that explains the difference in SRH levels.

The quality of the model for severity of depression as dependent variable is much better than for SRH (Table 5). The explanatory power of models explains up to 30% of the inter-individual variance when including controls (with already 8% explained by model 1 only). We equally note that the residual explanatory power is much higher and that this is mainly due to the variables included in model 1, meaning that our variables of interest influence directly the severity of depression over time. Even when controlling for indirect effects, we observe the significant effects (B = −1.33; 95% CI: −2.43, −0.23) that regularization has on depression. Similarly, we observe higher levels of PHQ-9 scores after the onset of the pandemic (wave 3) compared to previous waves. This effect is however independent of the regularization effect on mental health.

**Table 5:** Effect of regularization on depression score (PHQ-9) using hybrid models.

	PHQ-9 score	
	MODEL 1	MODEL 2
	B coeff (95% CI)	B coeff (95% CI)
Applicant (ref. undocumented) (within-individual effect)	−0.93 (−1.89, 0.04)	−0.77 (−1.74, 0.20)
Regularized (ref. undocumented) (within-individual effect)	−1.56 (−2.65, −0.46)**	−1.33 (−2.43, −0.23)*
Applicant (ref. undocumented) (between-individual effect)	−2.67 (−4.12, −1.22)***	−1.55 (−2.95, −0.15)*
Regularized (ref. undocumented) (between-individual effect)	−3.28 (−4.48, −2.08)***	−2.31 (−3.48, −1.14)***
Wave 1 (ref. Wave 3)	−1.08 (−1.69, −0.48)***	−1.04 (−1.65, −0.44)***
Wave 2 (ref. Wave 3)	−0.77 (−1.31, −0.23)**	−0.62 (−1.17, −0.08)*
Wave 4 (ref. Wave 3)	0.14 (−0.43, 0.71)	0.22 (−0.35, 0.79)
Women (ref. Men)		0.75 (−0.20, 1.70)
Age at baseline		−0.11 (−0.15, −0.06)***
African (ref. Latino American)		2.12 (0.39, 3.84)*
East Asian (ref. Latino American)		−0.53 (−1.50, 0.44)
Eastern European (ref. Latino American)		−2.19 (−3.91, −0.47)*
Secondary education (ref. Primary education)		0.27 (−1.11, 1.65)
Tertiary education (ref. Primary education)		0.56 (−0.98, 2.09)
Length of stay at baseline		0.02 (−0.07, 0.11)
Ability to face an unexpected bill (ref. No)		−0.88 (−1.44, −0.34)**
Number of working hours		−0.02 (−0.03, −0.00)*
Satisfaction with housing conditions (scale from 0 to 10)		−0.27 (−0.38, −0.16)***
Poor (very bad to sufficient) proficiency in French (ref. Good to Excellent)		−0.20 (−0.72, 0.33)
Partnership status (single) (ref. Married)		0.53 (−0.27, 1.33)
Partnership status (unmarried couple) (ref. Married)		−0.25 (−1.09, 0.59)
Nb. Children		0.07(-0.21, 0.35)
Observations (n, N)	387, 1304	387, 1304
R2 interindividual	0.081	0.3
R2 residual	0.014	0.02

***p < 0.001; **p < 0.01; *p < 0.05, Data source: Parchemins study.

In Model 2, we equally observe that older participants report lower depression scores and that migrants originating from Africa report higher scores compared to Latino Americans (B = 2.12; 95% CI: 0.39, 3.84). This finding can be a result of higher levels of discrimination that may impact mental health. Contrarily, migrants from eastern Europe report less depression, which may be due to the special situation of Eastern European migrants working first and foremost in stable employment in the construction sector in our case (B = −2.19; 95% CI: −3.91, −0.47). They face equally less barriers for visiting their home countries (Consoli et al., 2022). The ability to pay an unexpected bill also decreases depression scores (B = −0.88; 95% CI: −1.44, −0.34), as does more hours worked (B = −0.02; 95% CI: −0.03, −0.00) or a higher satisfaction with the housing situation (B = −0.27; 95% CI: −0.38, −0.16). The work-related and precarity related items influence not only mental health, but also SRH. Contrary to existing literature, there are no observable differences between men and women in our sample.

## 4 Discussion

We recall our aim to shed light on the influence of regularization on SRH and mental health in a longitudinal perspective. First, our findings suggest the absence of direct effects of regularization on SRH at the intra-individual level over time, contrary to our expectations. While regularized migrants show higher levels of SRH compared to undocumented migrants overall, we are not able to prove a direct effect of regularization on SRH, contradicting our first hypothesis.

However, our findings point to a positive effect of regularization on severity of depression, confirming our second hypothesis. This effect is a direct consequence of regularization and is stable after controlling for social and economic factors. Up-to our knowledge, it is the first time that a direct effect of regularization on mental health can be proved in a longitudinal perspective.

In line with our third hypothesis, that the COVID-19 pandemic led to a deterioration of mental health, but also that the effect of regularization on mental health is independent of the COVID-19 pandemic, data partially confirm what we expected. While it was not our initial aim to assess the effect of the COVID-19 pandemic on self-reported health and mental health among newly regularized and undocumented migrants, our data show that the pandemic had no influence on levels of SRH, but increased scores of depression among undocumented and regularized migrants. This is not surprising as the often-precarious living condition of migrants experienced additional pressure when different working sectors important to migrants, such as restauration, were closed. In fact, work-place related worries were a major source of concern (Duvoisin et al., 2022).

Our findings are consistent with other research that found positive effects of regularization on wellbeing and mental health (Patler & Pirtle, 2018), but we add that even in a longitudinal perspective and when controlling for alternative explanations of altered mental health, we observe that change in residence status is an important predictor of mental health. In line with the many changes migrants experience when getting regularized, we argue that improved mental health comes first and foremost from the relief from different sources of stress. Our results are in line with findings that found that restrictive government policies negatively influence mental health (Juárez et al., 2019). Thus, regularization relieves migrants from discrimination and stress induced by immigration policies (Torres et al., 2018).

As shown by our qualitative data (Consoli et al., 2022), migrants feel no longer the need to hide from state officials such as police officers or border patrols. In addition, access to new services, such as better medical access, and social protection can explain improved mental health. We argue that regularization takes the feeling of “being stuck in life” away and enable migrants to project themselves more positively into the future, and this immediately when entering the application procedure (Consoli et al., 2022).

Regarding controls, we find that older migrants show a lower PHQ-9 score which is in line with research showing that older people report lower stress levels (Graham & Ruiz Pozuelo, 2017). Similarly, the finding on the importance of the housing situation corroborates other research from Europe, as an unstable housing situation has negative effects on mental health (Andersson et al., 2018; Amerio et al., 2022). Our findings also confirm the importance of education levels, income and employment situation for SRH (Cullati et al., 2014). An additional aspect in our study might be that those already reporting better health are more likely to apply for regularization (Fakhoury et al., 2021).

The discrepancy between SRH and PHQ-9 is an important finding to be discussed. First, SRH values are rather high when considering the poor socioeconomic conditions of the participants and their level of chronic conditions, second, these values change little over time. SRH reports a more subjective evaluation of one's health than the PHQ-9 score, which constructs an index from several - still self-evaluated - items. SRH provides a holistic assessment of individual health, summarizing different components (Jylha, 2009). It has been shown that SRH is sensitive to context; against life expectancy gains, self-rated health in high-income countries has tended to decrease, a change attributed to increasing health expectations in the general population. In addition, research acknowledged that SRH does more importantly capture physical functioning rather than mental health (Mavaddat et al., 2011; Ahmad et al., 2014). The rather positive evaluation of SRH among our participants might reflect their lower aspirations towards health, as experienced in their country of origin and as a result of their limited access to healthcare. Second, it could be argued that participants focus their own health evaluations on its functional or somatic dimension: they have to be in good health to be able to work, which is the main motivation of their migration. The stable SRH rates observed here over time, despite COVID-19 effects, might thus reflect participants' consistently positive appreciations of their own health. Since they tend to rate it rather favorably, despite evidence of their health problems as measured by mental health and chronic conditions, it also means that an improvement due to regularization, or a deterioration due to COVID-19, is less likely. In other words, SRH rates could reflect undocumented and newly regularized migrants' long-standing aspirations for good health, independently of COVID-19 and of their chronic conditions. Nevertheless, the rather high levels of depression show that these groups clearly face adversity, and that regularization can alleviate stressors weighing on mental health.

In terms of limits, our findings may be specific to the Swiss context and the case of Geneva. In the Swiss context, we also insist on the obligation to subscribe to health assurance once regularized. Contrary to other countries, it is not possible to not subscribe, meaning that documented migrants face automatically health insurance costs contrary to other countries where the financial situation could lead to an avoidance of health insurance subscription. We add that groups under study (undocumented and newly regularized migrants in Geneva) are highly heterogenous and include migrants working in sectors such as construction, domestic work or restauration to mention a few, and with specific socio-demographics compared to other contexts. Still, our findings are well in line with earlier results, where different migrant groups show different mental health levels (Lindert et al., 2008; Andersson et al., 2018). However, our analysis is able to go beyond this result as we show that regularization has still a positive effect on mental health once controlling for belonging to different migrant groups or even for COVID-19-effects. By controlling for precarity-related variables, we are also able to show, that the effects are independent of the specific criteria for participation in the regularization scheme as income levels may have influenced health service consumption and thereby general health levels. At the end, we can however not exclude that the pandemic reduced possible positive short-term effects on SRH. In addition, participants do not include refugees nor short-term residents. Thus, all results from this study need to be repeated in other contexts, but we are confident that the results apply also to other regions and countries which share comparable economic and migration policies with Switzerland.

The findings have important consequences for policy formulation. Our results highlight positive effects of integrative immigration policies on mental health of undocumented migrants, but underscore equally that direct effects on SRH are less likely to be visible on the short term. In their systematic analysis, Vernice et al. (2020) showed that mental health is linked to the configuration of immigration policies and whether access to healthcare is possible. Similarly, Piccoli and Wanner (2022), emphasized that health of undocumented migrants differs between Swiss cantons depending on the level of inclusiveness of health policies. Our findings allow to nuance and point to the importance of taking stressors away from undocumented migrants. While special regularization programs such as the Papyrus Operation can be a solution to improve mental health, a stronger policy focus on mental health of undocumented migrants and targeted policy solutions can already improve their situation.

## 5 Conclusion

Our paper assessed what happens to self-reported health and mental health once undocumented migrants get regularized. Our findings, once controlling for alternative explanations as well as pandemic-related effects in hybrid models, are robust and show the positive effect of regularization itself on mental health only. For SRH, we find no direct effect of regularization on the short term, even though regularized migrants show higher levels of self-reported health. This finding appears to be in line with the argument that SRH summarizes multiple dimensions of health in a measure that accounts for individuals' circumstances across migration and aspirations. This does not exclude that SRH may change as a consequence of regularization on the long term, but we are not able to show a direct effect with our data. Further observations could shed light on the long-term consequences of regularization.

Consequently, our findings underscore the importance of regularization for mental health while questioning what exactly gets measured through SRH among regularized and undocumented migrants.

## Ethical statement

The study protocol was approved by the Ethics Committee of the Canton of Geneva, Switzerland (authorization 2017–00897). The data was collected through computer assisted interviews (CAPI). During the pandemic, data collection changed temporarily to videoconferencing. Participants gave informed consent at the beginning of the study and before every wave of data collection. An opportunity to opt-out was given at any moment and participants were regularly asked on their continued willingness to participate.

All persons who meet authorship criteria are listed as authors, and all authors certify that they have participated sufficiently in the work to take public responsibility for the content, including participation in the concept, design, analysis, writing, or revision of the manuscript.

## Declaration of competing interest

None.

## Data Availability

The data that has been used is confidential.
